# Commentary: Non-invasive Brain Stimulation, a Tool to Revert Maladaptive Plasticity in Neuropathic Pain

**DOI:** 10.3389/fnhum.2016.00544

**Published:** 2016-10-27

**Authors:** Marta Zantedeschi, Mariella Pazzaglia

**Affiliations:** ^1^Department of Psychology, University of Rome “La Sapienza”Rome, Italy; ^2^IRCCS Santa Lucia FoundationRome, Italy

**Keywords:** spinal cord injury, TMS, neuropathic pain, NIBS, plasticity, tDCS

Neuropathic pain (NP) is considered a definitive marker of maladaptive plasticity, which may be treated using non-invasive neuromodulatory techniques (Pascoal-Faria et al., 2015). In their stimulating and timely paper, Naro et al. (2016) review the significant progress made in studies of the use of non-invasive brain stimulation (NIBS) as an effective therapy to reduce chronic pain. The authors argued that NIBS interventions may induce neural changes that oppose the maladaptive plasticity at an early stage, thus representing a significant opportunity to prevent the development of NP.

As NP affects many individuals with spinal cord injury (SCI), this group may offer a model to test the above hypothesis. The reported prevalence of NP in SCI varies from 65–90% (Bonica, 1991; Siddall and Loeser, 2001; Siddall et al., 2003; Finnerup, 2013). The pain is severe and disabling in 18–63% of cases (Hagen and Rekand, 2015), has a devastating impact on quality of life (Margot-Duclot et al., 2009), and is often refractory to medical treatment. Pain in SCI often begins immediately after onset of injury (Siddall et al., 2003), with reports of up to 75% of cases presenting with early NP (Teixeira et al., 2013). Meanwhile, only 4–6% report any improvement (Siddall and Loeser, 2001).

Commonly, NP in SCI has been attributed to maladaptive plasticity (Costigan et al., 2009). Several studies have demonstrated that deefferentation and deafferentation lead to profound and long-lasting functional and structural cortical reorganization (Wrigley et al., 2009; Freund et al., 2011; Henderson et al., 2011). In SCI, there appear to be numerous, complex changes in several brain areas involved in body motor, sensorial, and nociceptive processing, including primary and sensory cortices, and the anterior cingulate cortex (Lotze et al., 1999, 2006; Kokotilo et al., 2009). Dysfunctional and abnormal reorganization may occur secondary to altered spino-thalamic and spino-cerebellar input, and presumably reflects the adaptation of extensive neural networks to altered body signals (Bruehlmeier et al., 1998). This phenomenon leads to an imbalance in the excitatory and inhibitory inputs to somatosensory and motor cortices, which could lead to a maladaptive reorganization associated with the development and maintenance of pain (Wrigley et al., 2009; Soler et al., 2010b). Indeed, a significant relationship has been observed between the degree of cortical reorganization and the intensity and duration of ongoing pain, which has led to the assumption that NP is invariably associated with somatosensory cortex reorganization following complete SCI (Wrigley et al., 2009; Gustin et al., 2010). As such, those individuals displaying significant alterations in bodily neuronal activity that translate into long-term structural changes in the brain are more likely to develop NP (Gustin et al., 2010).

Despite these findings, recent evidence supports the possibility of a different view. The presence of below-level NP has been associated with preserved structure and function of the primary somatosensory and motor cortices (Mole et al., 2014). Furthermore, the ongoing nociception is hypothesized to prevent the development of a neural signature of maladaptive plasticity (Jutzeler et al., 2016), as is observed in amputees (Makin et al., 2013). These data showing that pain *per se* is not associated with cortical plasticity, suggest that NIBS treatments aimed at reversing cortical reorganization should consider other factors. Indeed, independent of whether pain induces, causes, or prevents ongoing maladaptive plasticity, a relationship seems to exist between the extent of the deafferented body and brain changes, as there exist structural and functional differences between people with and without pain. The critical factor required for the cortex to undergo functional reorganization is a disruption of bodily perception caused by the constant absence/alteration of sensorimotor input and output (Lucci and Pazzaglia, 2015).

Considering that approximately one-third of individuals with SCI develop NP in the paralyzed body parts below the level of injury (Yezierski, 2005), the cortical representation of the body within the sensory motor areas targeted by NIBS may be a critical factor for pain treatment. Evidence for a link between anatomical brain changes, body distortion, and pain emerge in SCI and other persistent pain conditions (Lotze and Moseley, 2007). Patients with pain describe an abnormal perception of the deafferented limb (heavy, floating, enlarged, and mislocated), while reductions in pain appear to be coincident with normalization of body representation, suggesting the link may be bidirectional (Pazzaglia and Zantedeschi, 2016). We have previously found that in complete SCI interventions, the affected body part can modulate the body representation and have powerful effects on pain (Lenggenhager et al., 2013; Pazzaglia and Molinari, 2016; Pazzaglia et al., 2016). Such findings reveal the remarkable complexity and flexibility of body and pain plasticity in SCI, suggesting that a greater understanding of the cause of NP is necessary before any progress is made toward the application of the NIBS treatment.

Until now, isolated interventions applying both transcranial magnetic stimulation and transcranial direct current stimulation in SCI yielded conflicting results regarding the amelioration of pain. However, when NIBS and a visual illusion of the body were combined, the synergistic effects of the intervention produced better, and longer lasting analgesic benefits for reducing the overall severity of continuous NP compared with NIBS alone (Soler et al., 2010a).

Although the actual mechanisms underlying these effects remain to be elucidated, consideration of bodily perception opens the possibility of combined interventions with non-invasive procedures. These can be designed to preserve body representation and restore precise cortical topography, even when sensations are transferred to a different cortical territory. This may act as a means of preventing maladaptive plasticity and thereby prevent and manage refractory NP. The interplay between body awareness and pain as documented in visually-induced analgesia (Longo et al., 2012) bodily resizing (Mancini et al., 2011), allodynia (Pazzaglia et al., 2016), the use of virtual walking (Moseley, 2007; Soler et al., 2010a) and functional prostheses (Pazzaglia et al., 2013; Galli and Pazzaglia, 2015; Galli et al., 2015; Pazzaglia and Molinari, 2016) is imperative when considering current evidence supporting NIBS treatment, and will aid the rapid development of this potential rehabilitative therapy and the research supporting it. Clearly, more work is needed.

## Author contributions

MP and MZ work conception and design, work revision, final approval, global agreement.

### Conflict of interest statement

The authors declare that the research was conducted in the absence of any commercial or financial relationships that could be construed as a potential conflict of interest.
